# A novel stroke lesion network mapping approach: improved accuracy yet still low deficit prediction

**DOI:** 10.1093/braincomms/fcab259

**Published:** 2021-11-13

**Authors:** Lorenzo Pini, Alessandro Salvalaggio, Michele De Filippo De Grazia, Marco Zorzi, Michel Thiebaut de Schotten, Maurizio Corbetta

**Affiliations:** 1 Padova Neuroscience Center (PNC), University of Padova, Padova, 35100 Italy; 2 Clinica Neurologica, Department of Neuroscience, University of Padova, Padova, 35100 Italy; 3 IRCCS San Camillo Hospital, Venice, 30126 Italy; 4 Department of General Psychology, University of Padova, Padova, 35100 Italy; 5 Brain Connectivity and Behaviour Laboratory, Sorbonne Universities, Paris, 75006 France; 6 Groupe d’Imagerie Neurofonctionnelle, Institut des Maladies Neurodégénératives-UMR 5293, CNRS, CEA University of Bordeaux, Bordeaux, 33076 France; 7 Venetian Institute of Molecular Medicine, VIMM, Padova, 35100 Italy

**Keywords:** stroke, functional connectivity, behaviour, resting-state networks

## Abstract

Lesion network mapping estimates functional network abnormalities caused by a focal brain lesion. The method requires embedding the volume of the lesion into a normative functional connectome and using the average functional magnetic resonance imaging signal from that volume to compute the temporal correlation with all other brain locations. Lesion network mapping yields a map of potentially functionally disconnected regions. Although promising, this approach does not predict behavioural deficits well. We modified lesion network mapping by using the first principal component of the functional magnetic resonance imaging signal computed from the voxels within the lesioned area for temporal correlation. We measured potential improvements in connectivity strength, anatomical specificity of the lesioned network and behavioural prediction in a large cohort of first-time stroke patients at 2-weeks post-injury (*n* = 123). This principal component functional disconnection approach localized mainly cortical voxels of high signal-to-noise; and it yielded networks with higher anatomical specificity, and stronger behavioural correlation than the standard method. However, when examined with a rigorous leave-one-out machine learning approach, principal component functional disconnection approach did not perform better than the standard lesion network mapping in predicting neurological deficits. In summary, even though our novel method improves the specificity of disconnected networks and correlates with behavioural deficits post-stroke, it does not improve clinical prediction. Further work is needed to capture the complex adjustment of functional networks produced by focal damage in relation to behaviour.

## Teaser

Pini et al. developed a new methodology for ‘lesion network mapping’ in stroke. This new methodology enabled a more accurate estimation of functional brain network disconnected in stroke. However, the method did not predict well neurological impairment limiting its application to clinical questions.

## Graphical Abstract

**Graphical Abstract:**
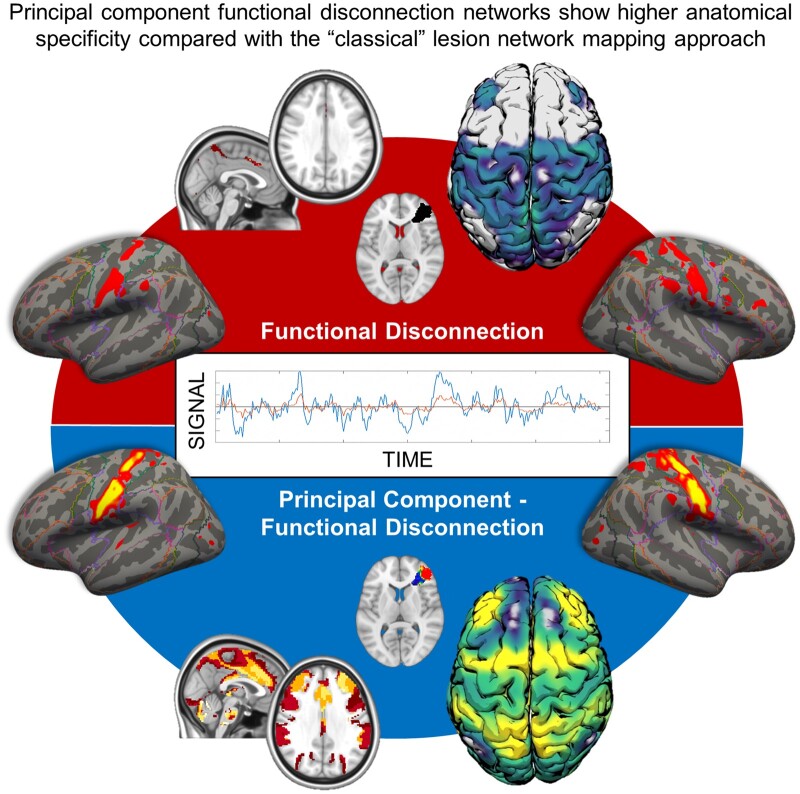

## Introduction

Pathological or traumatic events deeply affect the human brain’s functional architecture, triggering both local and distal reshaping of its intrinsic organization.^1–6^ These alterations can be estimated non-invasively through resting-state fMRI that measures the synchronization of the blood oxygenation level-dependent (BOLD) signal fluctuations between brain regions at rest. This functional connectivity (FC) proxy reveals the underlying structure of large-scale networks involved in cognitive and sensory processes,^7–10^ which exhibit selective vulnerability to several pathological events.

Strokes cause changes in the FC, specifically reducing inter-hemispheric connectivity and increasing intra-hemispheric connectivity between networks usually segregated (i.e. the default mode network—DMN—and the dorsal attention network) (for a review, see Corbetta et al.^11^). Notably, FC changes correlate with acute behavioural deficits and recovery.^12^^,^^13^ Thus, FC may be used, in principle, as a biomarker of recovery and to monitor therapy.^14^^,^^15^ However, robust FC analyses require significant scanning time (at least 15 min),^16^ are prone to artefacts (e.g. motion in the scanner), and require extensive pre-processing and statistical knowledge. As such, there is no key-ready package available in the clinical arena.

Recently, several methods have been introduced to estimate (indirectly) structural or functional network abnormalities from clinical scans. One of these methods, lesion network mapping (LNM), uses the topological information of lesions to derive binary seeds region-of-interest (ROIs) used to measure FC between the lesion and the rest of the brain.^17^ The resulting whole-brain functional maps are considered representative of the functional disconnection caused by the lesion on large-scale brain networks.^18^ This approach has been extensively used to describe network abnormalities of several conditions characterized by focal damage. More broadly, it is a general-purpose method to estimate functional network deficits from clinical scans.^17–22^

However, a debate recently flared up on the possible limitations of LMN.^23–28^ Some pitfalls regard statistical issues of sample size or thresholds applied to individual subject maps.^28^ Our group showed that LNM prediction of post-stroke behavioural deficits was significantly lower than lesion topography and indirect structural disconnection,^29^ a method similar to LNM but applied to diffusion tractography.^30^

There are several possibilities to explain LNM failure to predict post-stroke behavioural deficits. First, stroke lesions can affect physiology of distant regions through anatomical disconnection. In addition, they can cause alteration of functional interactions, especially between regions that are secondarily connected with the area of damage.^11^ LNM estimates regions and networks that are directly disconnected but does not consider changes in functional interactions of regions that are not directly linked to the lesion, e.g. through polysynaptic pathways. Recent studies show that these adjustments in functional interaction significantly contribute to behavioural dysfunction accounting for large proportions of variance across subjects.^4^^,^^11^^,^^13^^,^^31^ Second, stroke lesions might involve both grey (GM) and white matter (WM).^31^^,^^32^ WM BOLD signals are about ¼ to ½ lower amplitude than in GM,^33^^,^^34^ and can be drastically influenced by physiological noise.^35^ Therefore, since in LNM an averaged BOLD signal time-course is estimated from the whole lesion (both GM and WM), we and others have argued that the following whole-brain temporal correlation maps may be noisy and not anatomically accurate.^23^^,^^25^

Different approaches can be applied, such as masking with GM template.^36^^,^^37^ However, this procedure has several limitations. A simple mask would not consider the variability of BOLD signal. Moreover, lesions located entirely in the WM would be excluded from the analysis. This might be problematic in stroke, which is predominantly concentrated in subcortical and central WM.^31^^,^^38^ Moreover, although the functional role of WM BOLD signals remains controversial, preliminary evidence reported an intrinsic functional organization of WM linked with cognitive functions.^39^^,^^40^

To partially solve these issues, we present a modified principal component-functional disconnection (PC-FDC) method to estimate brain-wide disconnection from focal lesions. As in standard LNM, we embed each patient’s lesion in a normative healthy subject fMRI atlas dataset using the lesion as a ROI. However, rather than using the whole lesion, we first apply a principal component analysis (PCA) to the mean signal time-courses from each voxel within the lesion. This procedure selects only the voxels with the most similar time-course (first principal component, PC1). We compute whole-brain temporal correlation maps from these voxels, i.e. regions putatively functionally disconnected (PC-FDC map). In fMRI methodology, PCA has been successfully applied to identify robust spatial and temporal neural patterns free of artefacts.^35^^,^^41^

In this manuscript, we compare PC-FDC versus standard FDC (computed using time-courses from the whole lesion),^29^ similarly to LNM.^17^^,^^18^ These methods are compared in terms of the strength of the maps' temporal correlation, their anatomical specificity, and their ability to predict behavioural deficits 2-week post-stroke. We report that the new method yields maps with higher anatomical specificity, still, unfortunately, low behavioural prediction.

## Materials and methods

### Stroke sample and assessment

We retrospectively included 123 patients from the Washington University Stroke project (mean age 53 years; range 22–77; 119 right-handed; 63 females; 64 right hemispheres).^31^ First-time stroke patients were enrolled prospectively through the in-patient service at Barnes-Jewish Hospital and Rehabilitation Institute of St. Louis. All participants from this dataset provided written informed consent following the Declaration of Helsinki principles and procedures established by the Washington University in Saint Louis Institutional Review Board. Inclusion criteria were as follows: (i) age 18 or older; (ii) first symptomatic stroke, ischaemic or haemorrhagic; (iii) evidence of any neurological deficits; and (iv) time of enrolment <2 weeks post-stroke onset. Exclusion criteria were as follows: (i) inability to stay awake during testing; (ii) low (<1 y) life expectancy; (iii) evidence of clinically significant periventricular white matter disease; and (iv) a medical history of neurological, or psychiatric conditions that could interfere with the assessment plus contraindications for MRI.

Patients underwent a full MRI protocol and extensive cognitive assessment. Structural data were acquired on a 3 T Siemens Tim-Trio scanner (School of Medicine of the Washington University in St. Louis) consisting of a: sagittal MP-RAGE T_1_ weighted image (TR/TE: 1950/2.26 ms; flip angle: 9°; voxel size: 1 mm isotropic); transverse turbo spin-echo T_2_-weighted image (TR/TE: 2500/435 ms, voxel size: 1 mm isotropic); sagittal FLAIR (TR/TE: 7500/326 ms, voxel size: 1.5 mm isotropic).

The behavioural battery included 42 different measures covering the following domains: language, spatial memory, verbal memory, attention (the visual field bias), motor and visual (for details, see Corbetta et al.^31^). For the latter two domains (visual and motor), the scores were considered separately for left and right lesions. The battery lasted about 2.5 h and was performed within 24 h of MRI. Raw scores were normalized based on a control population (*n* = 31), and a PCA was run within each domain as in Corbetta et al.^31^ and Ramsey et al.^42^ The resulting component (factor) scores in each domain were used for the analysis as in our previous studies.^29^^,^^31^^,^^42^ For the language domain, data of *n* = 110 patients were available, while data for the attention and memory (both verbal and spatial) domains were available for *n* = 93 and *n* = 85 patients, respectively. Visual and motor left-right data were available for *n* = 48 and *n* = 23 patients, while scores for the right-left motor and visual domains were available for *n* = 54 and *n* = 29 patients, respectively.

### The principal component functional disconnection method

**Figure 1:**
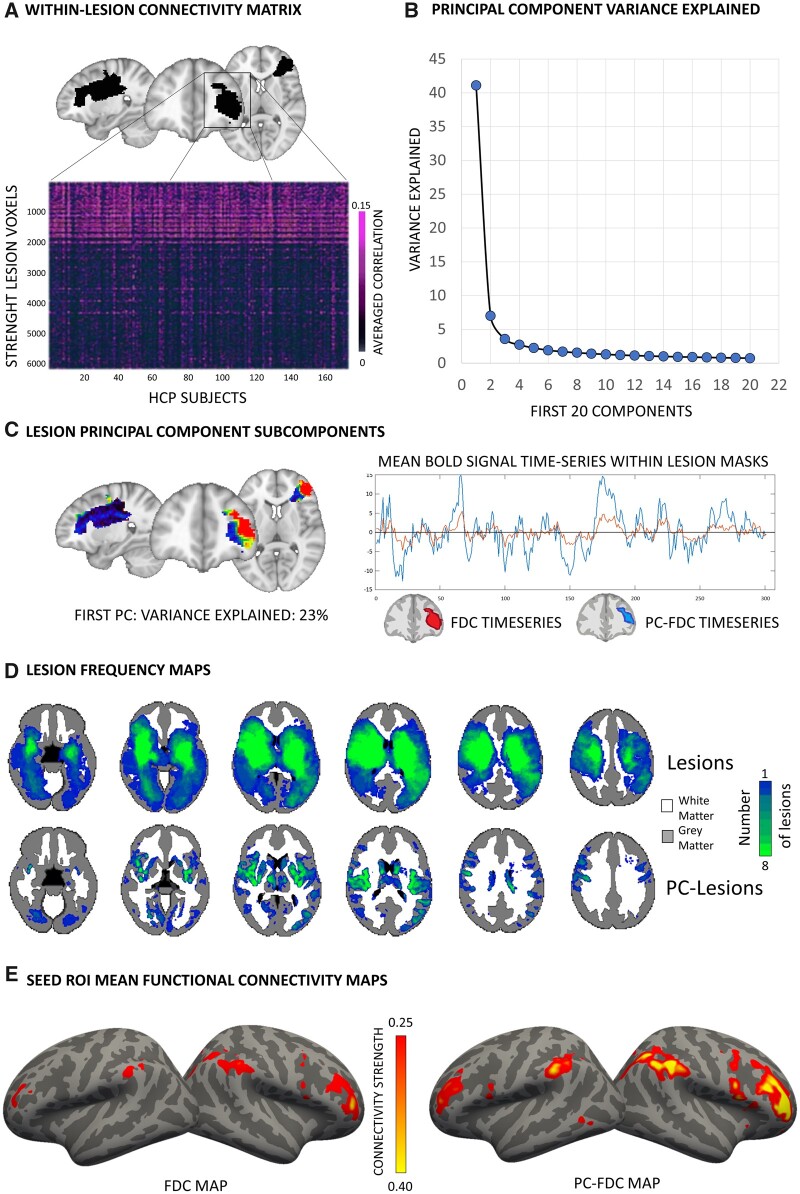
**Workflow of the PC-FDC analysis.** (**A**) Within-lesion (black mask overlaid in the MNI template) functional connectivity was computed indirectly in an independent sample of controls. (**B**) The voxel-averaged matrix was fed into a principal component analysis. (**C**) The first component was considered and projected back to lesion space. The time-series of the voxels overlapping with the 20% of the higher PC1-coefficient distribution was averaged and used to compute mean functional connectivity maps. (**D**) Lesion frequency maps. PC-FDC methodology identified lesion-voxels mainly in the GM. (**E**) Functional maps computed with the FDC and PC-FDC approaches were compared.

Brain lesions were manually segmented on individual structural MRI as reported in Siegel et al.^13^ and normalized to MNI through an enantiomorphic approach, which replaces lesioned tissue with the healthy contralateral tissue improving the quality of the registration, implemented in the BCBToolkit.^30^ Normalized lesions were resampled to 2×2×2 voxel space, binarized, and used as seed-ROIs for FC computation through the normative 7 T Human Connectome Project (HCP) dataset. We used the same functional data (*n* = 173) described in Salvalaggio et al.^29^ Lesion network maps were computed with both our PC-FDC and the FDC approach. For the FDC approach, whole-brain temporal correlation maps were calculated using the entire lesion as seed ROI by averaging the signal time-course across all voxels within the lesion. By contrast, the PC-FDC approach involved several steps to reduce the noise introduced by averaging signal time courses from different tissues within the lesion (e.g. WM and GM). First, we computed voxel-wise Pearson’s correlations among signal time courses within each lesion, resulting in *n* × *n* matrix (*n* = voxels affected by lesions). Correlation values were then *z*-Fisher transformed. Values of each row within this matrix were averaged, resulting in a vector of length *n*, representing each voxel's mean connectivity strength to the rest of the lesion. This procedure was repeated for every healthy control from the 7 T normative dataset. Finally, the averaged vectors were merged, resulting in a *n* × *m* (*m* = HCP subjects) matrix. The transpose *n*-by-*m* data matrix was then fed into the PCA algorithm. Subjects with a global absolute lesion-connectivity strength above 3 standard deviation from the sample lesion-connectivity mean were excluded from the lesion-PCA analysis to reduce PCA susceptibility to single brain outliers.^43^ As the PC1 explained the largest amount of variance, it was referred to as the main within-lesion connectivity axis. Subsequently, we projected back the coefficients to the lesion space (using the matrix of PC1 coefficients). The whole procedure was implemented through Matlab (Version 2019a). We considered only voxels with absolute coefficients higher than the 20th percentile of the distribution. This threshold was used to identify along the PC1 gradient the subset of voxels above that threshold. These new lesion-PC1 binarized maps were then used as seed-ROI to compute whole-brain temporal correlation (Pearson *r*) maps in the normative HCP sample.^17^^,^^29^ This procedure was independently repeated for every lesion of our dataset (123 lesions). The procedure failed for six lesions, due to slight misalignments, thus leaving a total of 117 lesion maps for subsequent analysis. For a visualization of the methodology, see Fig.  1.

For both methods (PC-FDC and FDC) and each lesion, the resulting lesion network maps were *z*-Fisher transformed and averaged (r-maps). Additionally, we computed the t-maps through a one-sample *t*-test implement in fsl randomise with *n* = 5000 permutations, as in Boes et al.^17^ While r-maps represent the average correlation between the lesion and the rest of the brain, t-maps express how much each voxel varies from the mean across single maps.

### Statistical analysis

#### Indirect functional connectivity strength

We compared differences in FC strength between the lesion and the rest of the brain computed with both approaches (i.e. PC-FDC versus FDC). The Wilcoxon signed-ranks test implemented in Matlab (Version 2019a) was used to compare the distributions of global mean (only *z*-Fisher positive) FC values. Moreover, we compared mean FC for higher values (i.e. *z-*Fisher threshold > 0.2). As complementary analysis, Wilcoxon signed-ranks test was used to compare the standard deviation values of each lesion network map computed with FDC and PC-FDC. This analysis, together with the mean analysis, investigated whether our methodology can generate maps with higher sensitivity, i.e. higher mean and equal/lower standard deviation than the standard method.

Additionally, we investigated differences in voxel-wise FC values distribution. To this aim, we first computed the cumulative distribution function (CDF) of every r-map at the voxel level (only positive value, masking zero values). We then compared voxel values between PC-FDC and FDC for different cut-offs of the CDF (10th, 20th, 50th, 80th, 90th, 95th and 99th) to capture the whole spectrum of voxel correlational values distribution. Values distribution for each cut-off between PC-FDC and FDC was compared with the Wilcoxon signed-ranks test. This analysis was performed at the whole-brain level and considering GM (cortical and subcortical) and WM masks from the Harvard-Oxford atlas.

Finally, we compared connectivity values for lesions mainly overlapping with the WM. Pure WM lesions were identified computing the ratio of voxels falling in the WM and GM mask (from the Harvard-Oxford atlas). Lesions with a WM/size ratio greater than 0.9 were considered for this additional analysis. The Wilcoxon signed-ranks test was used to compare connectivity values between FDC and PC-FDC.

#### Network spatial organization

To investigate the anatomical precision of lesion network maps, we compared the spatial correlation between lesion network maps (for both PC-FDC and FDC approaches) with canonical networks template through the FSL utility *fslcc* (FSL v.6.0.0; https://fsl.fmrib.ox.ac.uk/fsl/). For each connectivity map, we computed a network confidence index (NCI) assignment based on a winner-take-all approach as follows:

$$\mathrm{NCI}=\frac{sc.\mathrm{CNet}(a)}{\Sigma_{k=1}^{n}sc.\mathrm{CNet}\left(k\right)/n}$$

where *sc* refers to the spatial correlation of each lesion networks with a canonical template (*CNet), a* marks the canonical template with the highest correlation (winner template), and *k* represents the other canonical templates (losing templates). Higher NCI represents a proxy of the degree of a lesion network's spatial specificity with the large-scale canonical organization reported in the literature. The Wilcoxon signed-ranks test was used to compare the NCI between the PC-FDC and the FDC approaches. NCI was computed using different atlases to evaluate whether other templates might substantially influence results. To this aim, we used the network maps reported by Yeo et al.^9^ and Shirer et al.^44^ We excluded the limbic network from the Yeo et al.^9^ atlas from this analysis due to the high susceptibility to signal distortions of its core regions, the orbitofrontal and medial temporal lobe.^45^^,^^46^ From Shirer’s template,^44^ the precuneus network was not included in the NCI computation, as partially overlapping with the dorsal and ventral DMN atlases merged into a unique DMN mask. Finally, NCI was computed independently for r-maps and t-maps for comparison purposes.

#### The relationship between lesion network maps and motor deficits

To further explore the two approaches' anatomical specificity, we investigated network strength differences at the voxel-wise level. Owing to the importance of motor symptoms in ischaemic stroke,^47^ we *a priori* focussed this analysis in the motor domain.

Specifically, we selected a subset of patients with motor performance lower than 2 standard deviation (from a healthy control distribution; for details, see Corbetta et al.^31^). Network r-maps (thresholded at *z*-Fisher > 0.2) from lesions in both hemispheres were considered and pooled together. Voxel-wise differences between PC-FDC and FDC were investigated through a paired nonparametric inference based on *fsl randomise* with *n* = 5000 permutations. Multiple comparisons were corrected across space using a familywise error (FWE) based on permutation testing at a threshold-free cluster enhancement (TFCE). We tested the following contrasts: PC-FDC > FDC; FDC > PC-FDC. Significance was set at a *P*-value < 0.025 FWE-corrected, corresponding to a two-tailed *P* < 0.05. Finally, the Wilcoxon signed-ranks test contrasted the NCI statistically for FDC and PC-FDC, considering the sensorimotor network (SMN) as the target network, rather than using a winner-tale-all approach.

We further compared the network topography in patients falling within the lowest (<20th percentile distribution of motor scores) and the highest motor score distribution (>80th percentile distribution of motor performance). This cut-off was chosen as trade-off between the motor performance and a sample size robust enough to perform voxel-wise comparisons. We considered both patients with right and left lesions. Differences between lesion network maps (r-maps *z*-Fisher > 0.2) computed with the two approaches were investigated at voxel-wise level using the same model reported above with *fsl randomise*.

We further performed an explorative voxel-wise analysis comparing lesion maps from stroke patients with the highest (>80th percentile) and lowest (<20th percentile) performance in the language domain. The same model was implemented aimed at investigating whether spatial improvement linked with the PC-FDC approach was observed also in a cognitive domain.

#### Behavioural-connectivity association

We further compared the relationship between FC and behavioural performance to assess whether PC-FDC maps showed a stronger association with deficits. We computed the *r* Pearson’s correlation between behavioural performance and global mean connectivity (r-maps un-thresholded positive) for each behavioural domain. The same analysis was repeated covarying the lesion size. Results were corrected for multiple comparisons (*n* = 8 behavioural domains) with Bonferroni correction, and *P*-value < 0.006 was considered significant.

This association was also investigated at the voxel-wise level. Specifically, we implemented a simple linear correlation through nonparametric inference using *fsl randomise* (*n* = 5000 permutations FWE-corrected at TFCE). The analysis was corrected for lesion size, using the volume of lesions as covariate. For each behavioural domain, univariate maps expressing the significant association were considered at a stringent significant *P*-values < 0.01 FWE-corrected. Univariate maps were thresholded and binarized (at the significant *P*-values) to compute the agreement between FDC, PC-FDC and canonical (Yeo’s) template using the dice coefficient. Higher dice overlap reflects higher overlap between two maps, thus better agreement.

#### Behavioural-connectivity prediction

A multivariate analysis was carried out using r-maps features as predictors and behavioural scores for each domain as outcome variables. Our approach used a ridge regression (RR) model that uses L2-normalization to regularize coefficients to preventing overfitting and improving generalization on test data.^48^ To reduce the input dimensionality, the features of the individual r-maps were extracted by PCA and used as multivariate predictors to predict patients’ behavioural outcomes.^13^^,^^29^ PCA was performed on 902’229 2-mm^3^ brain voxels and returns a set of PC scores. All PC scores were *z*-normalized based on the mean and variance of the whole PC matrix. Finally, PC that explained 95% of the variance were retained and used as input in the ridge regression model. The model weights *W* are computed as:

$$W={\left(X^{T}X+\lambda I\right)}^{-1}X^{T}Y$$

where *X* is the PC matrix and *Y* is the *z*-normalized outcome variable. The regularization term $\lambda \left(\mathrm{lambda}\right)$ provides a constraint on the size of the weights. To find the appropriate value of λ a tuning procedure was carried out using a leave-one-(patient)-out cross validation loop (LOOCV). This procedure allows to train and test the better ridge regression models respect the regularization term. In each loop λ was optimized through 100 values in range 10^−5^,10^5^ (logarithmic step) to minimize the leave-one-out prediction error over the training set. Optimal weights were solved using gradient descent to minimize error for the ridge regression equation by varying lambda. These weights were then used to predict the outcome variable for the left-out test patient. The whole prediction was generated for all patients in this way. Model accuracy was assessed using the coefficient of determination

$$R^{2}=1-\frac{{\sum \left(Y-Y'\right)}^{2}}{{\sum \left(Y-\overset{'}{Y'}\right)}^{2}}$$

where Y are the measured outcome variables, Y' are the predicted outcome variables and $\overset{'}{Y'}$ is the mean of Y'. For each model, the statistical significance was assessed using a permutation test. The outcome variables were randomly permuted across subjects 10 000 times. The entire regression process was carried out with each set of randomized labels. *P*-values were calculated as the probability of observing the reported $R^{2}$ values by chance (number of permutations *R*^2^ > observed *R*^2^/number of permutations). Only models with *P*-values < 0.05 were investigated.

The final set of ridge regression weights was generated by averaging the weight matrix across all n LOOCV loops. Each final weight's statistical reliability was assessed by comparing its distribution of values to a null distribution (null models generated for permutation testing) using an FDR corrected *t*-test. The final set of statistically reliable weights was back projected to the brain (using the transpose matrix of PC coefficients) to create a 3D map of the most predictive voxels.

#### Sensitivity analysis

We investigated PC-FDC outcomes computed with different coefficient thresholds, using the 5th, the 10th, the 20th, the 50th and 80th coefficients percentile. To this aim, we compared connectivity strength between network-maps for the highest cut-off of the CDF, as described in section "Indirect functional connectivity strength". Friedman test was used to investigate significant differences across connectivity values.

### Data availability

The functional dataset used for this analysis is publicly available from the Human Connectome Project (http://humanconnectome.org/). Stroke lesions and behavioural data can be accessed at http://cnda.wustl.edu/app/template/Login.vm. PC-FDC and statistical maps can be downloaded at https://github.com/pinilorenzo/PC-FDC_maps. Data are also available via request to the corresponding author.

## Results

### Summary of method

Figure  1 shows the highlights of the revised method for lesion network mapping (PC-FDC) vis-à-vis the traditional method (FDC). A PCA is run on a matrix composed of lesion voxels × subjects on the BOLD time-series (Fig.  1A). In all lesions, the PC1 explains a significant fraction of the variance (Fig.  1B). The weights of PC1 are then back projected onto the original lesion (Fig.  1C). This procedure separates GM from WM, with the highest loadings in the GM (as shown in the frequency maps of Fig.  1D). This is explained by the higher signal-to-noise of the GM BOLD signal as compared to WM.^33^ The corresponding BOLD signal time series from PC1 lesion ROI (PC-FDC) have higher amplitude as compared to the signal time course obtained from averaging across the whole ROI (FDC) (inset Fig.  1C). The resulting lesion network maps show a stronger connectivity profile (Fig.  1E).

### Lesion network strength and spatial coherence

**Figure 2:**
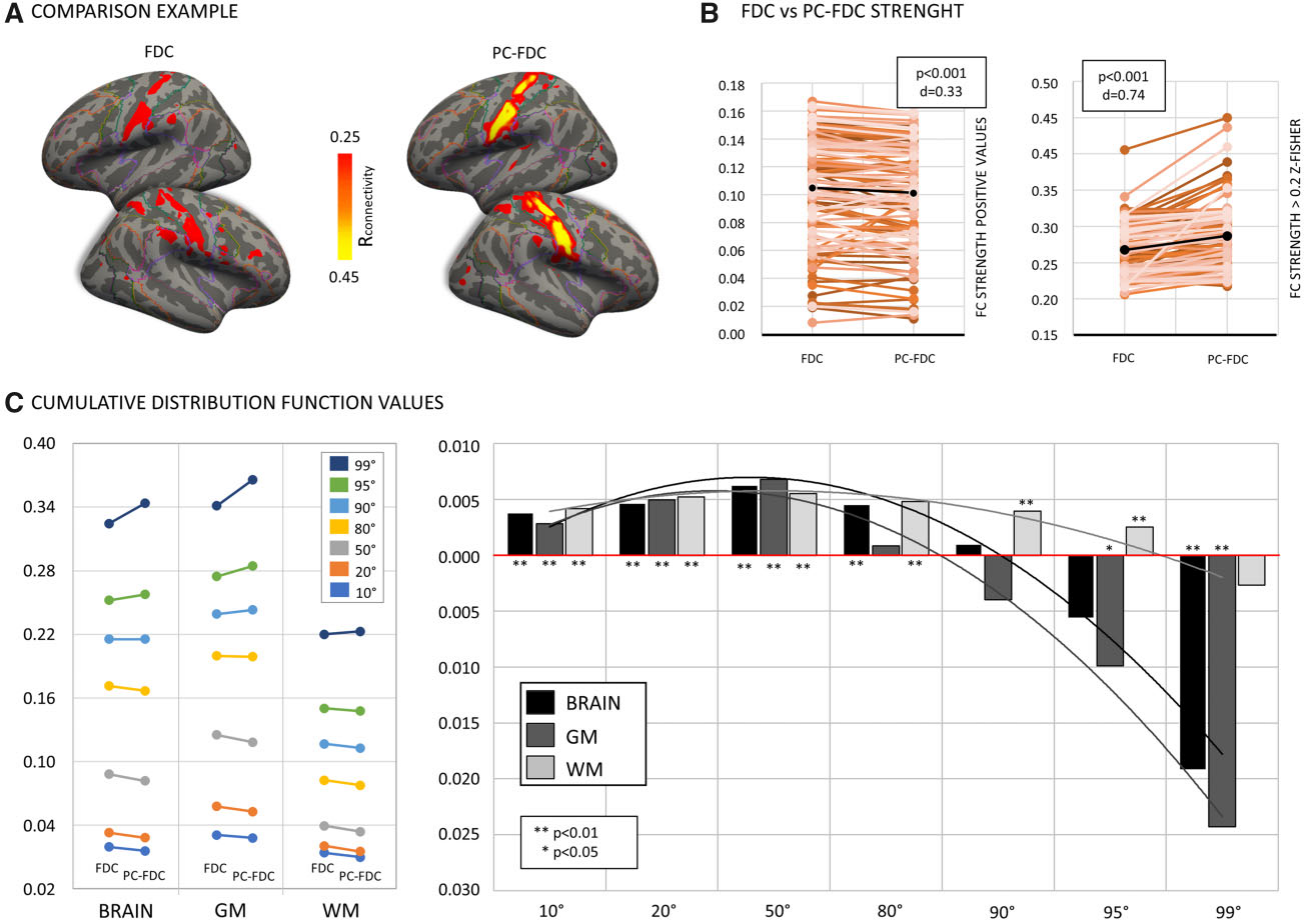
**Functional connectivity strength**. (**A**) Representative PC-FDC and FDC network-maps overlapping with the sensory-motor network. (**B**) Connectivity networks strength difference between PC-FDC and FDC for positive values (left) and *z*-Fisher > 0.2 (right). (**C**) Left panel shows connectivity values associated at different cumulative distribution function cut-offs for the FDC and the PC-FDC; right panel reports values as difference between FDC and PC-FDC connectivity maps [values are reported for the whole brain (black), grey matter (dark-grey) and white matter (light-grey) masks]. The Wilcoxon signed-ranks test was used to compare the connectivity profile between FDC and PC-FDC maps. *P*-values (*P*) and Cohen’s effect size (*d*) are reported.

Lesion-network maps computed with the two approaches were different, with PC-FDC showing stronger functional maps and higher anatomical specificity (Figs  1E and 2A show two representative maps classified as corresponding to the SMN and the frontoparietal network, respectively).

While the overall FC mean values of FDC maps were stronger when considering the whole range of positive values (*Z* = −4.867, *P* < 0.001, *d* = 0.33), the opposite was true when considering maps thresholded at *z*-Fisher > 0.2 (*Z* = 7.992, *P* < 0.001, *d* = 0.74), i.e. the strongest functional connections (Fig.  2B). This pattern is explained by the CDF analysis in which we plotted the same results at different thresholds of correlation (Fig.  2C). While FDC yielded higher connectivity values from most of the range (*P* < 0.01 for 10–80th percentile cut-offs), an inversion occurred with PC-FDC yielding higher mean FC after the 95th percentile cut-off, (95–99th percentile, *P* < 0. 01). Interestingly, while the FDC method showed significantly stronger FC with WM, as compared to GM voxels, for most of the range (*P* < 0.01 for all but not-significant for the 99th percentile cut-off), PC-FDC favoured GM significantly for the strongest connections (95th cut-off, *P* < 0.05). Finally, the standard deviation of lesion network maps generated through our new methodology was slightly decreased compared with FDC (*Z* = −2.890; *P* < 0.004).

For the WM lesion analysis, we identified 11 lesions falling in the WM (range ratio WM size/lesion size 0.91–1). For these lesions, PC-FDC connectivity values were significantly higher compared to the ‘classical’ FDC maps (*Z* = 7.231; *P* < 0.001; *d* = 1.04) (Supplementary Fig. 1).

**Figure 3:**
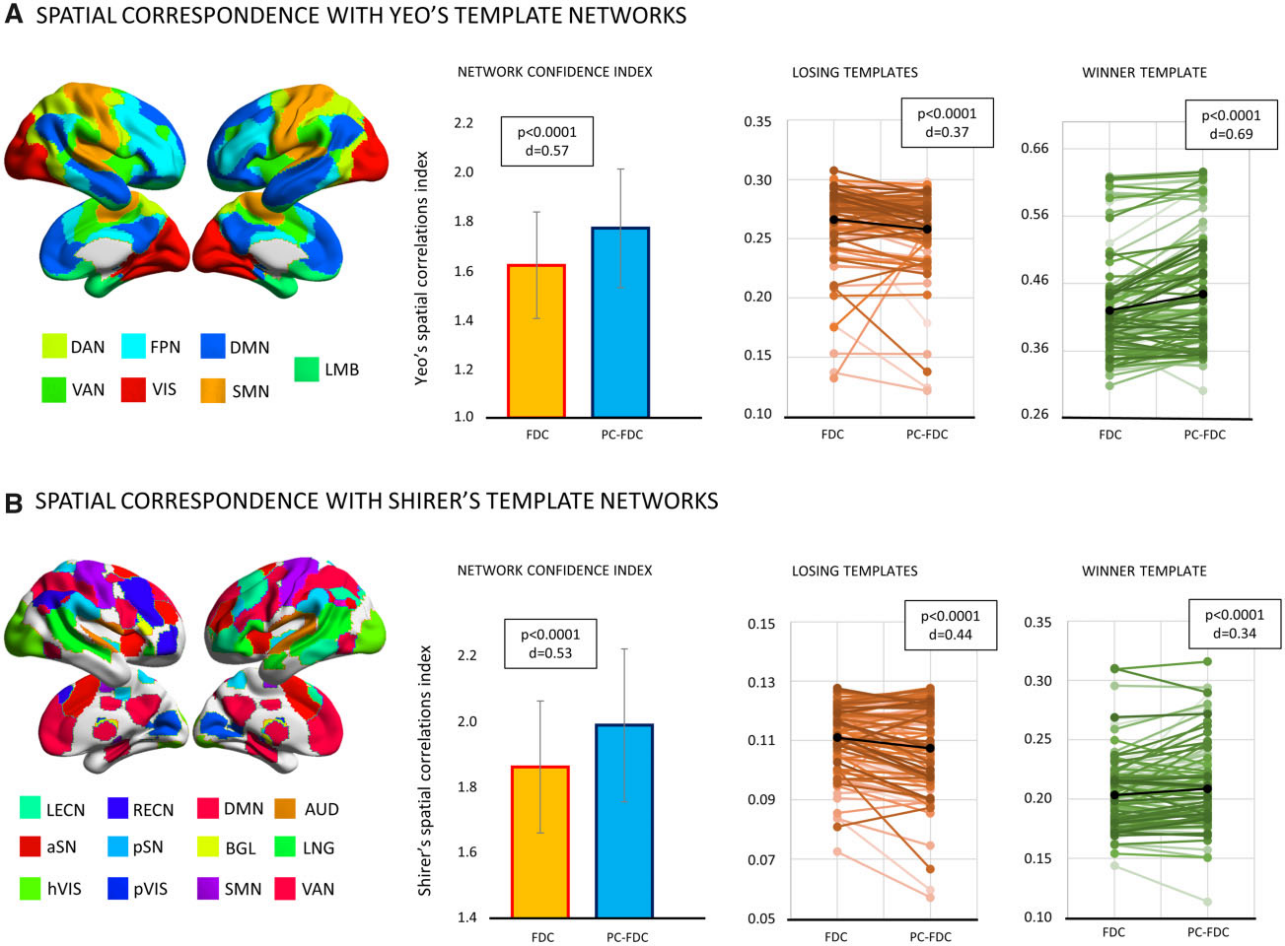
**Functional spatial specificity**. Comparison of the network confidence index, winner-template, and losing-templates spatial correlations between PC-FDC and FDC. These metrics were reported for Yeo’s (top) and Shirer’s (bottom) templates.^9^^,^^44^ The Wilcoxon signed-rank test was applied to compare network indices between FDC and PC-FDC. *P*-values (*P*) and Cohen’s effect size (*d*) are reported.

In the second analysis, we compared the anatomical specificity of PC-FDC versus FDC maps by measuring their spatial similarity to canonical brain networks derived from normative healthy subject atlases, specifically Yeo et al.^9^ and Shirer et al.^44^ To measure this similarity, we used the NCI (network correlation index) that weights both the similarity with the most similar atlas network (winner-template) and the dissimilarity with the other networks (losing-template) (see methods). The PC-FDC approach showed significantly higher NCI, hence higher overlap with canonical networks, independently of the atlas employed (Yeo *Z* = 6.373, *P* < 0.0001, *d* = 0.57; Shirer *Z* = 5.330, *P* < 0.0001, *d* = 0.53). This result reflected both a lower overlap with the losing-templates (Yeo *Z* = −5.651, *P* < 0.0001, *d* = 0.37; Shirer: *Z* = −4.370; *P* < 0.0001; *d* = 0.44) and a higher overlap with the winner-template (Yeo *Z* = 6.364, *P* < 0.0001, *d* = 0.69; Shirer: *Z* = 3.348; *P* < 0.0001; *d* = 0.34) (Fig.  3). This pattern was echoed using T-maps (positive values) (Supplementary Fig. 2).

### Connectivity lesion maps and motor domain

**Figure 4:**
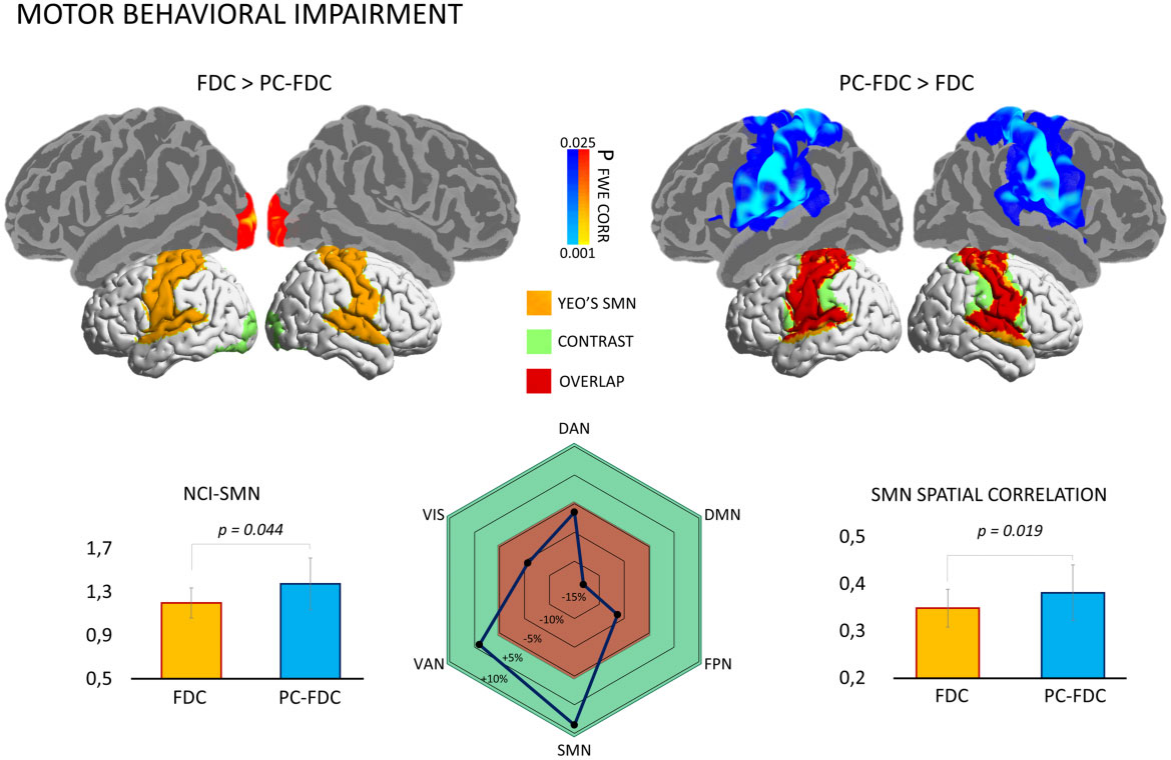
**Voxel-wise differences between FDC and PC-FDC network-maps**. *Top panel*: connectivity maps from patients with the lowest motor performance were compared at voxel-wise level (using the threshold-free cluster enhancement approach with *n* = 5000 permutation and statistical threshold set at *P* < 0.025 FWE-corrected). Significant voxels in the PC-FDC > FDC contrast (blue) and FDC > PC-FDC (red-yellow) were registered and overlaid in the fsaverage surface (top-panels). Significant maps were binarized and overlaid onto the brain surface with the Yeo’s motor network mask for comparison purpose. *Bottom panel*: The Wilcoxon signed-rank test was applied to compare both confidence index (left) and the spatial specificity (right) of the sensorimotor network (from Yeo et al.^9^) for both PC-FDC and FDC network-maps; The delta of the spatial correlation between FDC and PC-FDC maps with canonical templates is shown in the centre panel. DAN = dorsal-attention network; DMN = default mode network; FPN = frontoparietal network; SMN = sensorimotor network; VAN = ventral-attention network; VIS = visual network.

The previous analysis showed that the PC-FDC method yields an overall higher spatial match with healthy canonical networks than the standard FDC method. To test the two methods' anatomical specificity in relation to selective deficits, we compared FDC and PC-FDC lesion network maps from patients with motor impairment. Specifically, we pooled 9 patients with right motor deficits and 8 patients with left deficits for this analysis. PC-FDC maps compared to FDC showed stronger FC along the lateral and medial bilateral motor regions, overlapping with Yeo’s SMN (Fig.  4, top panel). Additionally, the spatial correlation with Yeo’s SMN and the corresponding NCI-SMN confidence index in this subsample were higher for the PC-FDC approach than for the FDC (SMN: *Z* = 2.343, *P* = 0.019; SMN-NCI: *Z* = 2.012, *P* = 0.044) (Fig.  4, bottom panel). Among networks, PC-FDC maps showed an increased spatial correlation with SMN (delta +10%), while the other Yeo’s templates showed a reduction of correlation (delta −5/−15%), except for ventral-attention network (delta +5%) (Fig.  4, bottom panel).

**Figure 5:**
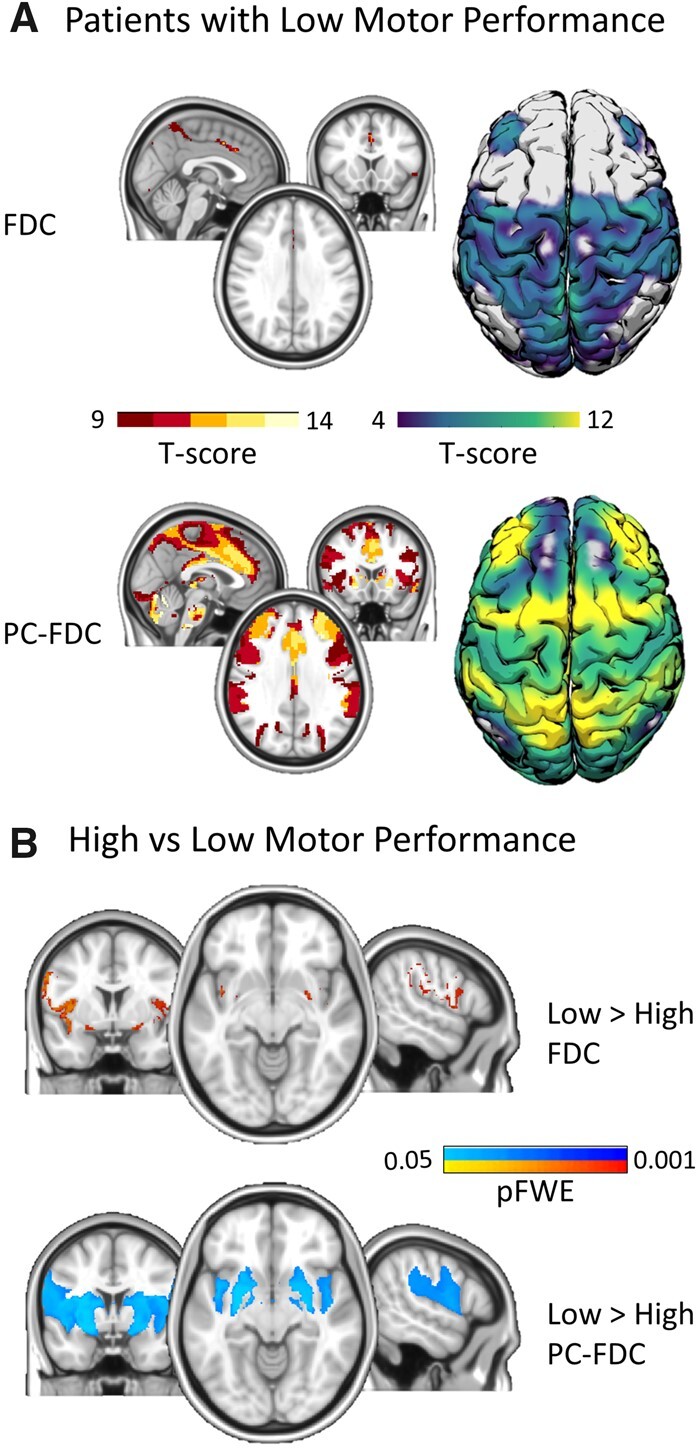
**Differences in patients with lower versus higher motor performance.** (**A)** One-sample *t*-test maps [threshold-free cluster enhancement (TFCE) approach with *n* = 5000 permutation], for patient with the lowest motor performance computed separately for the FDC (*top panel*) and the PC-FDC (*bottom panel*) approaches. **(B**) Two-sample *t*-test for the comparison between patients with motor impairment versus unimpaired (TFCE approach; *n* = 5000 permutation; *P* < 0.05 FWE-corrected). Motor impaired patients showed voxels with stronger dysconnectivity, more extended in the PC-FDC approach.

Patients with the lowest motor performance were compared with patients showing the highest motor scores (*n* = 21; 11 for right deficits and 10 for left deficits for each group; range motor *Z*-score: lowest group from −1.95 to −2.87; highest group from 0.63 to 1.19). Congruently with previous analysis, PC-FDC yielded stronger connectivity maps (see Fig.  5A). Specifically, PC-FDC networks showed higher connectivity values in a network including primary motor system, parietal, frontal and temporal cortices.

When we compared patient groups separately for the two methodologies, PC-FDC maps showed more extended dysconnectivity encompassing the striatum, insula, precentral gyrus and supramarginal gyrus at *P* < 0.05 FWE-corrected. By contrast, only few voxels were significantly different for the FDC application (Fig.  5B). The opposite contrast (lowest deficits > highest deficits) showed no significant results for both approaches.

A second analysis was carried out in a subset of patients with language performance at the two extremes of the behavioural distribution. For this analysis, we included 22 patients for each group (range language *Z*-score: low performance group: −0.52 to −3.25; high performance group: 0.65 to 0.94). Both approaches showed stronger disconnection in language impaired individuals (low > high performance) in two left frontal clusters involved in language abilities, namely the middle frontal gyrus and the pars opercularis. Additionally, the PC-FDC approach captured a disconnected cluster in the left superior parietal cortex (Supplementary Fig. 3). No significant differences were observed for the opposite contrast (high > low performance). This analysis suggested that our approach can improve the spatial topology of network disconnection for both motor and cognitive deficits.

### Lesion network and domain-specific behavioural performance

After establishing that PC-FDC yields more anatomically specific maps than standard FDC, we examined the correlation with behavioural performance. In an overall analysis, we found a negative association between language, spatial memory, and attention visual field scores with global mean connectivity surviving multiple comparisons (*P* < 0.006) for the PC-FDC approach. However, the correlation with the attention visual field did not survive after covarying for lesion size. By contrast, the FDC method produced only a significant correlation with visual right-left field deficits (Supplementary Fig. 4).

**Figure 6:**
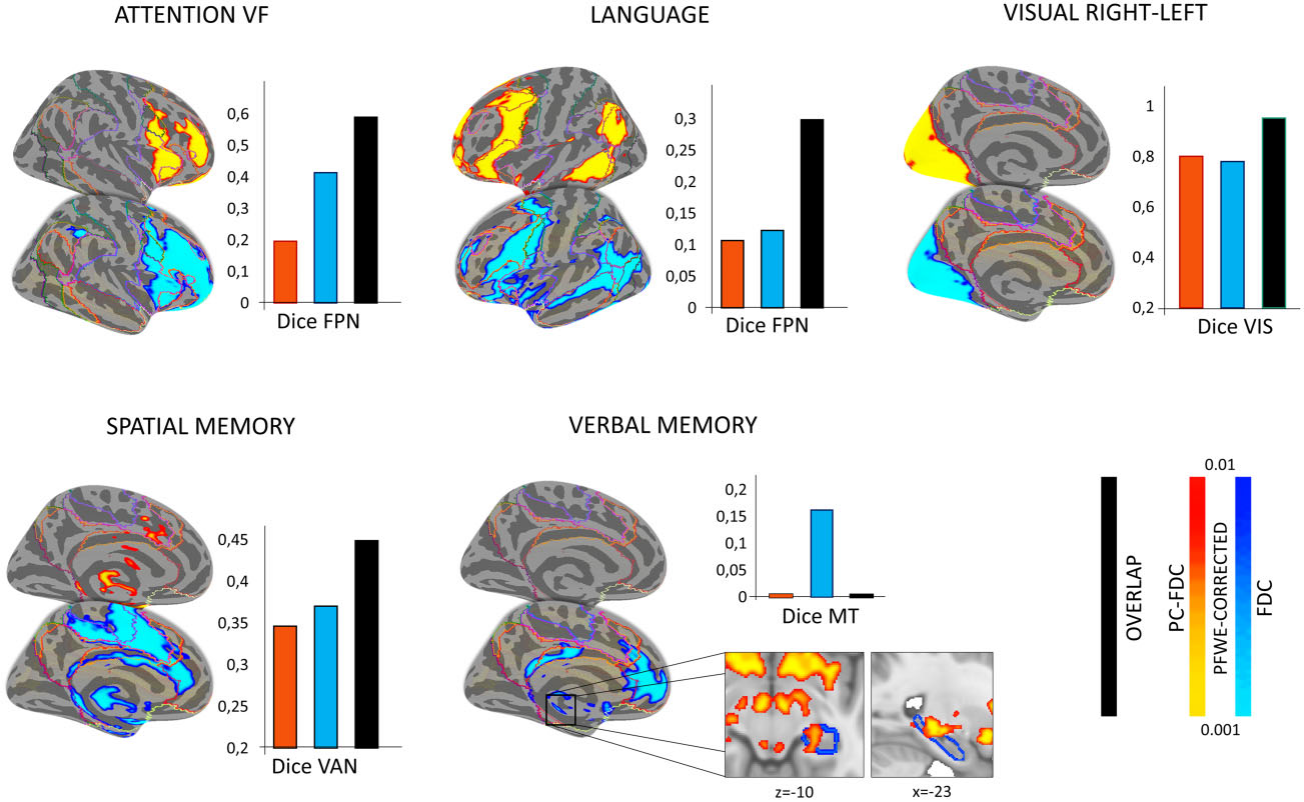
**Voxel-wise correlation between connectivity and behaviour.** Univariate maps from the PC-FDC (blue) and FDC (red) approach showing clusters linked with behaviour (simple linear correlation through nonparametric threshold-free cluster enhancement approach; *n* = 5000 permutation) covarying for lesion size (volume). Results are co-registered to the fsaverage surface and showed at pFWE < 0.01. For each significant univariate map, the corresponding dice coefficient with canonical template is showed (inset bar plots). FPN = frontoparietal network; MT = memory network; VAN = ventral-attention network; VIS = visual network. Lines outline Yeo’s 7 networks subdivision.

The univariate analysis (expressing the association between connectivity and behaviour at the voxel level) was in line with the above correlation pattern. Clusters from PC-FDC maps surviving the significance threshold were reported for all the cognitive domains and the visual right-left deficits. In contrast, FDC clusters for the verbal memory domain did not survive statistical significance (Fig.  6). Moreover, voxels from PC-FDC maps linked with cognitive domains showed a higher spatial consistency with canonical networks (expressed as dice coefficient): (i) frontoparietal network-attention, PFDC: *d* = 0.415 versus FDC: *d* = 0.197; frontoparietal network-language, PFDC: *d* = 0.123 versus FDC: *d* = 0.107; ventral-attention network-spatial memory, PC-FDC: *d* = 0.370 versus FDC: *d* = 0.346. The verbal memory domain showed significant PC-FDC clusters located in the retrosplenial-hippocampal network (see inset Fig.  6). This map partially overlapped with the memory circuity reported in Ferguson et al.^22^ (*d* = 0.162). Furthermore, using a less stringent statistical threshold (*P* < 0.05 FWE-corrected) the PC-FDC motor and visual maps (left-right deficits) showed higher spatial consistency with canonical networks (visual network-deficits, PFDC: *d* = 0.631 versus FDC: *d* = 0.337; motor network-deficits, PFDC: *d* = 0.40 versus FDC: *d* = 0.181) (Supplementary Fig. 5). By contrast, for both approaches the univariate map for the motor right-left deficits was not significant also with the lower threshold.

### Behavioural prediction

We were interested in re-examining whether this modified method was predictive of behavioural performance since, in previous work, we reported that the FDC method was not.^29^ For this purpose, we ran a ridge regression analysis to finds the lesion network maps that best explain behavioural variability. We investigated the predictive connectivity pattern for cognitive and visual-motor domains. For cognition (attention, language, memory), the prediction of connectivity maps computed with the PC-FDC approach was very low (*R*^2^ < 0.1 for all cognitive domains; Table  1). These values were comparable with the FDC approach reporting similar *R*^2^ < 0.1 results. Slightly higher results were reported for visual and motor domains, ranging from 0.08 for motor right-left to 0.55 for visual right-left (Table  1). Motor and visual left-right deficits were similar with FDC, while visual right-left showed higher predictive values (delta *R*^2^ +0.16). Predictive maps can be downloaded at https://github.com/pinilorenzo/PC-FDC_maps.

**Table 1:** Ridge regression behavioural prediction comparison from PC-FDC, FDC and structural lesion maps

	Patients	C (95%)	*R* ^2^	C (95%)	*R* ^2^	C (95%)	*R* ^2^	C (95%)	*R* ^2^
		FDC		PC-FDC		PC-Lesion		PC-Lesion + PC-FDC	
Language	110	6	0.03	7	0.05^*^	62	0.43^*^	69	0.54^*^
Attention VF	93	7	0.09^*^	8	0.08^*^	53	0.11^*^	61	0.12^*^
Memory Verbal	85	6	0.03	7	0.03	46	0.03^*^	53	0.09^*^
Memory Spatial	85	6	0.06^*^	7	0.09^*^	46	0.10^*^	53	0.11^*^
Motor L-R	48	5	0.16^*^	6	0.20^*^	26	0.25^*^	32	0.24^*^
Motor R-L	54	6	0.10^*^	6	0.08^*^	30	0.31^*^	36	0.29^*^
Visual L-R	23	4	0.14^*^	5	0.18^*^	23	0.42^*^	17	0.40^*^
Visual R-L	29	6	0.39^*^	7	0.55^*^	29	0.59^*^	24	overfit

C = number of components; L = left; R = right; VF = visual fields.

* Model significant.

However, predictive scores from connectivity were lower than predictive values from the structural map of the lesion reshaped after PCA (PC1 lesion map; Table  1) for all behavioural domains. The analysis using PC-FDC and PC1 lesion maps in combination leads to comparable results, except for the language domain (Table  1). The corresponding predictive maps showed both commonalities and differences with the univariate maps. Specifically, maps from cognitive domains showed the weakest spatial correspondence between ridge regression and univariate statistics. In contrast, visual-motor deficits showed the highest spatial correspondence (cognitive _mean_*d* = 0.22 versus visual-motor _mean_*d* = 0.59) (Supplementary Fig. 6).

Finally, we applied the ridge regression to PC-FDC and structural lesion maps computed with the second principal component (PC2). As for PC1, FC prediction was lower for all the domains considered compared to structural lesion maps (*R*^2^ < 0.1; Table  2). Notably, we reported divergent effects when we compared PC2 versus PC1 maps. Specifically, PC2 FC maps were less predictive in both cognitive (delta −5%) and sensory-motor (delta −10%) domains. This pattern was inverted when we considered structural maps, that is, PC2 lesion maps showed higher prediction in cognitive domains (delta +7%), albeit in the sensory-motor domain, this improvement was lowest (delta +3%).

**Table 2:** Ridge regression results from the second PC for functional and structural lesion maps

	Patients	C (95%)	*R* ^2^	C (95%)	*R* ^2^
		PC2-FDC		PC2-Lesion	
Language	110	7	0.00	64	0.41^*^
Attention VF	93	8	0.02	55	0.24^*^
Memory Verbal	85	7	0.01	47	0.06
Memory Spatial	85	7	0.04	47	0.25^*^
Motor L-R	48	6	0.18^*^	27	0.25^*^
Motor R-L	54	6	0.11^*^	31	0.36^*^
Visual L-R	23	5	0.06	23	0.44^*^
Visual R-L	29	7	0.26^*^	29	0.62^*^

C = number of components; L = left; PC2 = second PC; R = right; VF = visual fields.

* Model significant.

### Sensitivity analysis

We explored connectivity maps computed using different cut-offs for PC1 coefficients. PC-FDC computed using different PC-coefficient percentiles showed significant differences for the CDF cut-off considered [90th-CDF X2(4) = 214; *P* < 0.001; 95th-CDF X2(4) = 156; *P* < 0.001; 99th-CDF X2(4) = 91; *P* < 0.001]. *Posthoc* analysis revealed that maps computed with the 20th coefficient threshold showed stronger FC values compared to the other thresholds (*P* < 0.02; Supplementary Fig. 7).

Finally, PC1 explained a high amount of variance in our stroke sample compared to PC2 (mean variance: 41 ± 10 versus 7 ± 5). Moreover, PC1 was more representative of the cortical network mainly affected by a specific lesion. In the example reported in Supplementary Fig. 8, PC1 maps to the right frontal cortex, resulting in a cortical network exhibiting higher connectivity values. In contrast, the network computed with PC2 (whose highest coefficients mainly overlapped in the WM) showed lower strength. This lower correlation was evident for positive and anti-correlated values (Supplementary Fig. 8, Panel B).

## Discussion

We investigated whether a modified FDC (LNM) approach improves anatomical specificity and behavioural prediction post-stroke. We implemented a PCA approach to account for the variability of within-lesion connectivity patterns. This methodology enabled to derive a lesion subcomponent explaining within-connectivity maximal variance. Lesion reshaped based on PC1 was used to recompute whole-brain functional disconnection patterns. Compared to the FDC approach, PC-FDC maps showed a more biologically plausible pattern and higher anatomical specificity. However, the low behavioural prediction was comparable to the FDC method previously reported.^29^

In the following discussion, we first consider the merits of the method, its anatomical specificity, and behavioural prediction.

Most stroke lesions are small, the great majority hit both GM and WM, and tend to concentrate in the WM and basal ganglia. Pure cortical lesions are less than 20% of the total.^31^ These properties may affect the signal-to-noise of LNM when applied to stroke, given the signal time course from the lesion reflect a combination of GM, WM and CSF signals.^23^^,^^25^ PCA solves this problem by selecting more homogeneous signals, and we computed more biologically plausible cortical networks than the standard approach. Reshaping the lesion based on the similarity of the signal within it enabled the identification of voxels—represented in PC1—that showed the most robust functional connectivity, hence putative disconnection patterns.

Lesion maps computed with our methodology showed higher functional specificity, i.e. increased spatial correspondence with networks from the literature.^9^^,^^44^ Furthermore, PC-FDC and FDC showed a divergent pattern for lower and higher connectivity values. At low thresholds, i.e. including most voxels in the lesion network map, FDC yielded higher overall FC values that localized, however, both to WM and GM. In contrast, the PC-FDC method produced stronger connectivity at high thresholds highlighting only the strongest functional connections, which localized almost exclusively to GM. Therefore, the PC-FDC approach improves the signal-to-noise of lesion-network maps by denoising the signal used for the computation (see time courses for PC-FDC and FDC in Fig.  1). Similarly, for stroke lesions located mainly in the WM, our methodology generated stronger WM functional maps by suppressing noise, in line with previous studies investigating WM networks.^39^

The PC-FDC method was anatomically more specific when investigating motor deficits. Compared with FDC maps, the PC-FDC approach yielded higher correspondence with motor maps.^9^ Specifically, PC-FDC connectivity clusters compared to the classical approach enabled to compute voxels with higher connectivity within the M1, supplementary motor area, and ventral and dorsal premotor cortices, overlapping with the canonical SMN map. When we investigated lesion network maps in patients with motor impairment versus patients without motor impairment, PC-FDC maps revealed a stronger and more extended dysconnectivity pattern in the former group. Similarly, in an explorative analysis on language deficits we found stronger disconnection with the PC-FDC method. Although both FDC and PC-FDC showed comparable clusters of dysconnectivity in the middle frontal gyrus and the pars opercularis, regions involved in language abilities,^49^^,^^50^ only PC-FDC localized additional disconnection in the left superior parietal region, which has been suggested as a key language area.^51^ Therefore, the PC-FDC method improves anatomical specificity of disconnected networks, the prime application of LNM.^17^^,^^18^

Next, we consider the correlation with behaviour. The novel method showed a higher correlation than the standard method with cognitive scores in univariate regression analyses. PC-FDC maps significantly correlated with language and spatial memory scores after multiple comparison correction with lesion size as a covariate. Moreover, verbal memory scores were associated with disconnection of the retro-splenial cortex and hippocampus, but only in PC-FDC maps. This map partially overlaps with the memory circuit from Ferguson et al.^22^ (see inset Fig.  5B). Similarly, the topological relationship between connectivity with motor and visual maps was improved by the PC-FDC methodology.

However, when testing behavioural prediction using machine learning ridge-regression, the PC-FDC method did not perform better than the standard approach. PC-FDC maps explained around 6% of the variance, more than ten percentage points below the variance explained by the structural lesions reshaped through PC1.

Why does indirect functional disconnection method fail to predict cognitive deficits? The main limitation is that the method assumes that behavioural deficits depend on functionally disconnected regions. However, both directly and indirectly anatomically disconnected regions produce a complex modification of multi-network connectivity patterns, even for small lesions.^11^^,^^13^ These distributed connectivity changes will not show up in indirect methods. In fact, lesions cause both a decrease in within-network integration and a decrease in between-network segregation, leading to an overall decrement of modularity.^4^^,^^52^^,^^53^ Moreover, brain connections are unevenly distributed, with some regions more densely interconnected than others.^54^ The effect of lesions on brain networks is enhanced when the damage hits GM or WM hubs.^4^^,^^31^^,^^55^ Thus, the different levels of functional hierarchical re-organization induced by lesions are not apparent in LNM.

In contrast to cognitive deficits, PC-FDC maps showed slightly higher prediction, ranging from 8% to 20% for motor and 18% to 55% for visual deficits, still lower than structural lesions. A possible reason for the higher predictability of sensory and motor deficits is the intrinsic organization of functional connections. The association between structure and function progressively diverges, moving from unimodal (i.e. sensory) to trans-modal (i.e. associative) cortices.^56^ The trans-modal regions' organization might allow a more flexible and integrated response to different types of stimuli.^57^ Accordingly, these regions' alterations propagate upstream and downstream through connectors (regions of integration between modules) or rich club hubs with a cascade effect on cognitive abilities.^58^ By contrast, sensory and motor networks show a higher synchronization level within the same circuit, and their activity is strongly dependent on inputs.^16^^,^^59^ Thus, lesions within these circuits might lead to more circumscribed functional disconnection effects that can be more easily predicted with indirect methods.

This study has both strengths and limitations. The main strength is that we applied a PCA to reduce noise contribution in the connectivity maps computed from lesions. For this analysis, we only considered eigenvalues with higher values from the PC1. Moreover, we applied different analysis levels to study connectivity specificity, strength, behavioural relationship, and prediction through different state-of-the-art techniques. Although we analysed different thresholds for PC1, showing that the 20th percentile allowed the highest functional specificity and strength, we cannot rule out the possibility that a better refinement of the threshold might increase the prediction results. Moreover, we did not compare this new approach with a simple masking procedure. Further studies should evaluate behavioural prediction of GM/WM masking procedures. Finally, the low number of components for indirect FC measures might be linked with poor behavioural prediction. Thus, the consideration of more components and the application of non-linear dimensionality reduction techniques in future studies building on this first attempt should shed light on whether we can improve behavioural prediction for indirect functional measures. Finally, future studies should assess whether different machine learning algorithms might increase the post-stroke behavioural prediction of indirect functional outcomes.

## Conclusions

In conclusion, a PCA approach improved the anatomical specificity and the strength of estimated functionally disconnected networks from focal lesions. Despite higher signal-to-noise and improved correlation with behavioural deficits, predominantly sensory and motor, we could not obtain precise out-of-sample predictions with indirect functional measures. Owing to the complex nature of the interaction between cognitive process and brain FC, direct estimation of FC connectivity seems essential to building a more precise and reliable behavioural deficits model following stroke lesion.

## Supplementary material

Supplementary material is available at *Brain Communications* online.

## Funding

M.C. was supported by Flagship ERA-NET Joint Transnational Call 2017 (grant ANR-17-HBPR-0001); Italian Ministero dell'Istruzione—Ministero dell'Università e della Ricerca (MIUR)—Departments of Excellence Italian Ministry of Research (MART_ECCELLENZA18_01); Fondazione Cassa di Risparmio di Padova e Rovigo (CARIPARO)—Ricerca Scientifica di Eccellenza 2018 (Grant Agreement number 55403); Italian Ministero della Salute, Brain connectivity measured with high-density electroencephalography: a novel neurodiagnostic tool for stroke (NEUROCONN; RF-2008-12366899); Celeghin Foundation Padova (CUP C94I20000420007); BIAL foundation grant (No. 361/18); Horizon 2020 European School of Network Neuroscience—European School of Network Neuroscience (euSNN), H2020-SC5-2019-2 (Grant Agreement number 869505); Horizon 2020 research and innovation programme; Visionary Nature Based Actions For Heath, Wellbeing & Resilience in Cities (VARCITIES), Horizon 2020-SC5-2019-2 (Grant Agreement number 869505); Italian Ministero della Salute: Eye-movement dynamics during free viewing as biomarker for assessment of visuospatial functions and for closed-loop rehabilitation in stroke (EYEMOVINSTROKE; RF-2019-12369300); M.T.d.S. has received funding from the European Research Council (ERC) under the European Union’s Horizon 2020 research and innovation programme (grant agreement no. 818521).

## Competing interests

The authors report no competing interests.

## Supplementary Material

fcab259_Supplementary_Data

## Abbreviations

BOLD =: blood oxygenation level-dependent
CDF =: cumulative distribution function
DMN =: default mode network
FC =: functional connectivity
FWE =: familywise error
GM =: grey matter
HCP =: Human Connectome Project
LNM =: lesion network mapping
NCI =: network confidence index
PCA =: principal component analysis
PC-FDC =: principal component-functional disconnection
ROI =: region-of-interest
RR =: ridge regression
SMN =: sensorimotor network
WM =: white matter
