# Supplementary material for: A novel stroke lesion network mapping approach: improved accuracy yet still low deficit prediction
Source: Brain Commun. 2021 Nov 13;3(4):fcab259. doi: 10.1093/braincomms/fcab259 (PMC8633453; doi:10.1093/braincomms/fcab259)
Supplement: fcab259_Supplementary_Data [file fcab259_supplementary_data.docx]

**Supplementary Figure S1. Connectivity maps from lesions mainly located in the white matter.**

Panel A: frequency map of a subset of lesions mainly located in the white matter (n=11); Panel B: difference in brain connectivity values between PC-FDC and FDC functional maps. For these lesions, the Wilcoxon signed-ranks test showed significant white matter (WM) stronger values for PC-FDC maps compared to FDC. P-values (p) and Cohen’s effect size (d) are reported.

**
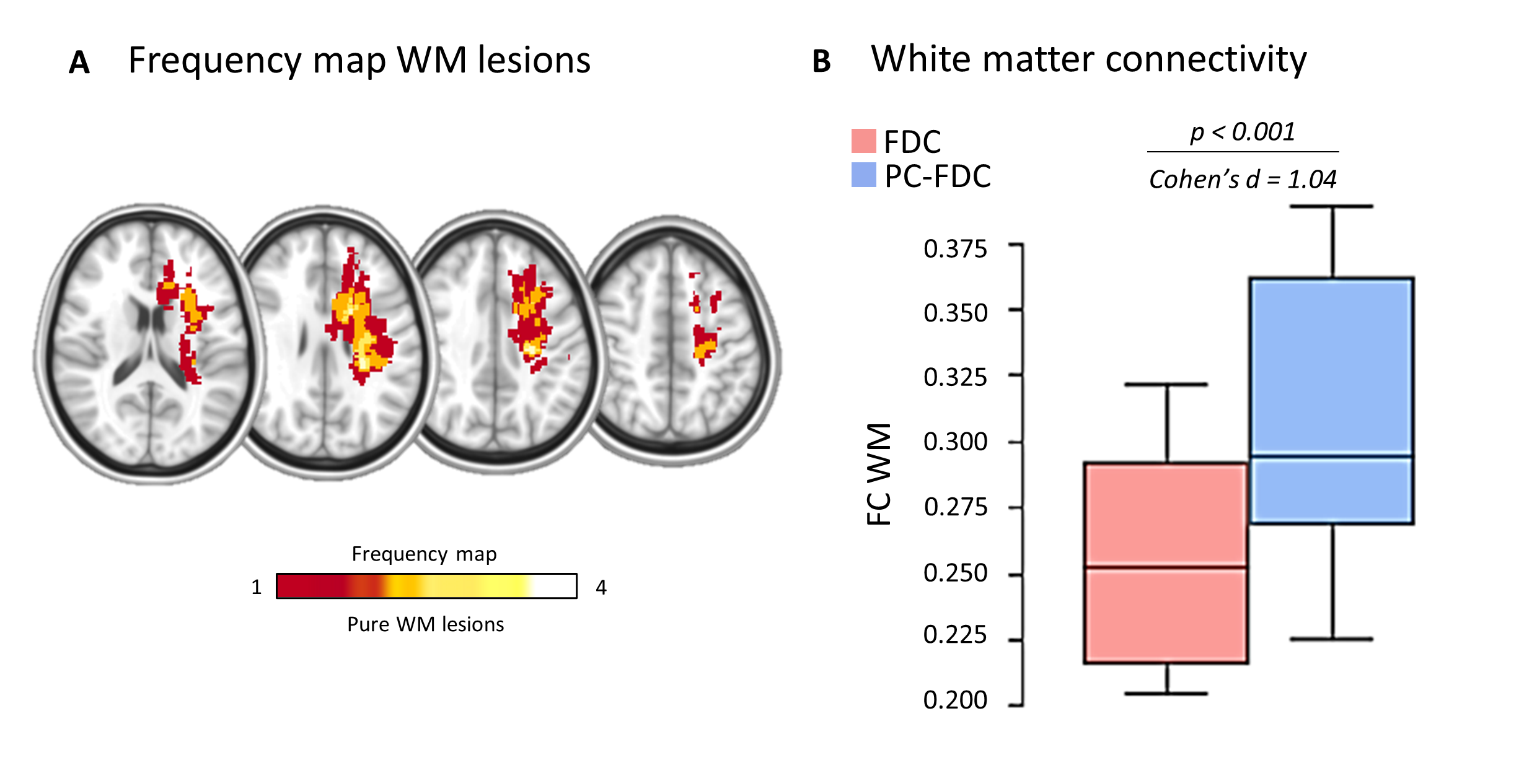
**

**Supplementary Figure S2. Functional spatial specificity of T-maps.**

Comparison of the network confidence index, winner-template and losing-templates spatial correlations between PC-FDC and FDC for (positive) t-maps. These metrics were reported for Yeo’s (top) and Shirer’s (bottom) template. The Wilcoxon signed-rank test was applied to compare network indices between FDC and PC-FDC. P-values (p) and Cohen’s effect size (d) are reported.


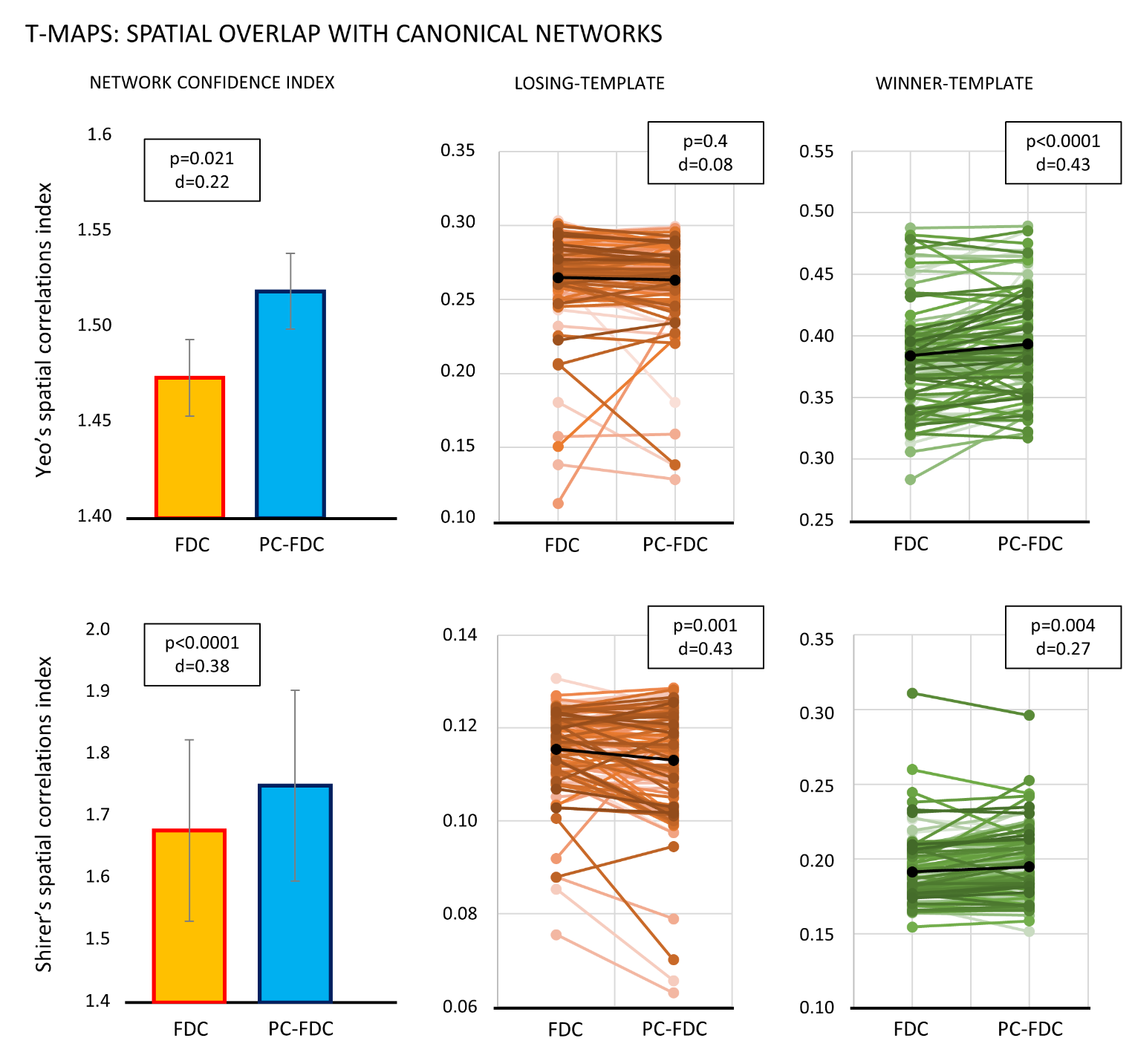


**Supplementary Figure S3. Differences in patients with lower vs. higher language performance.**

Two-sample t-test for the comparison between patients with language impairment vs language unimpaired (computed with the threshold-free cluster enhancement approach; n=5000 permutation; p<0.05 FWE-corrected). Both approaches showed left frontal dysconnectivity in patients relying in the lowest language score distribution compared to patients with highest scores. PC-FDC methodology identified an additional disconnected cluster in the left superior parietal gyrus. L: left; R: right.

**
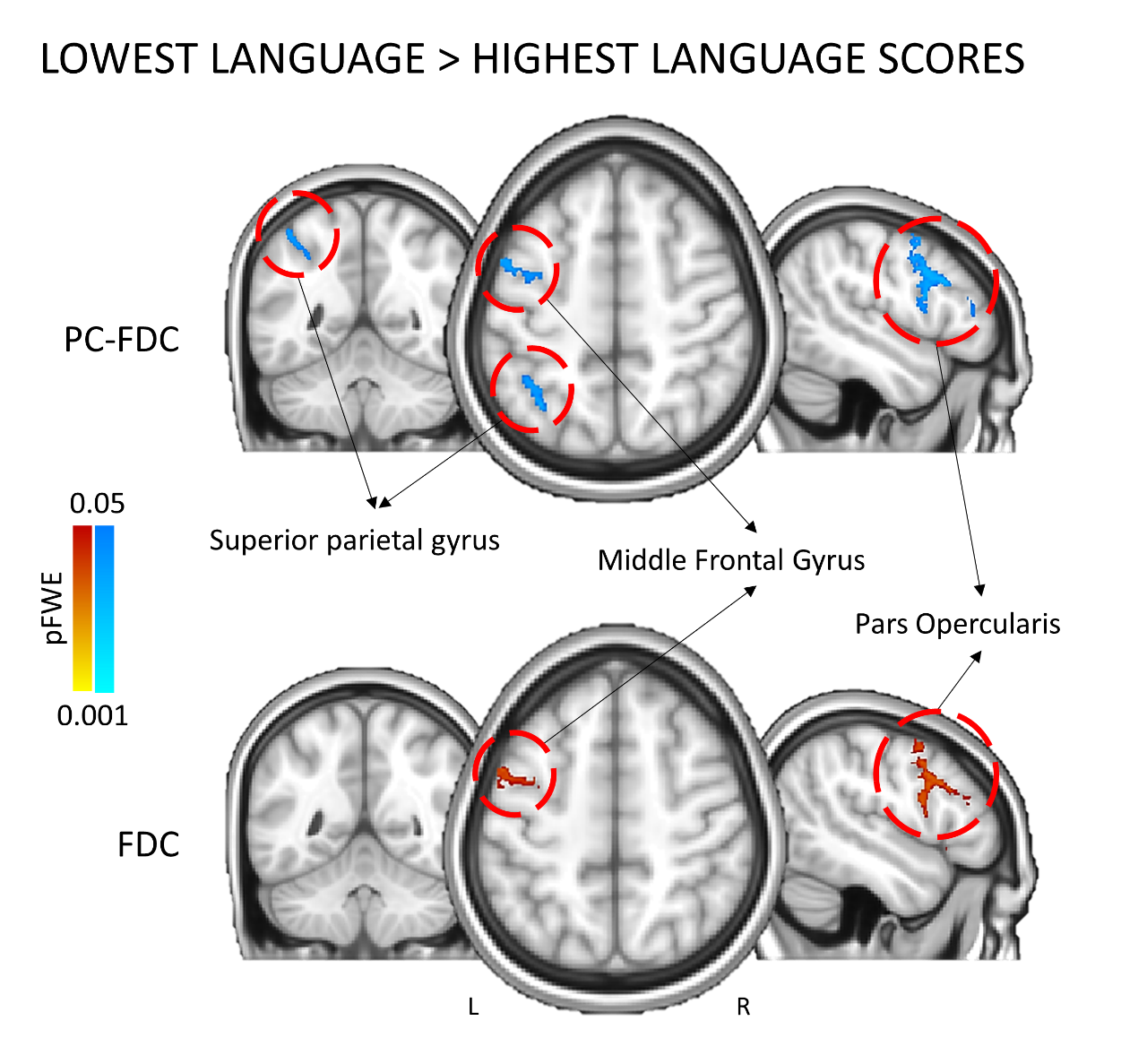
**

**Supplementary Figure S4.** **Behavior-connectivity coupling in stroke patients.**

Correlation (r Pearson’s) between mean connectivity strength and behavioral scores for both FDC and PC-FDC approaches. * marks significant correlations surviving multiple comparisons (p<0.006); # marks significant correlation after covarying for lesion size.

**
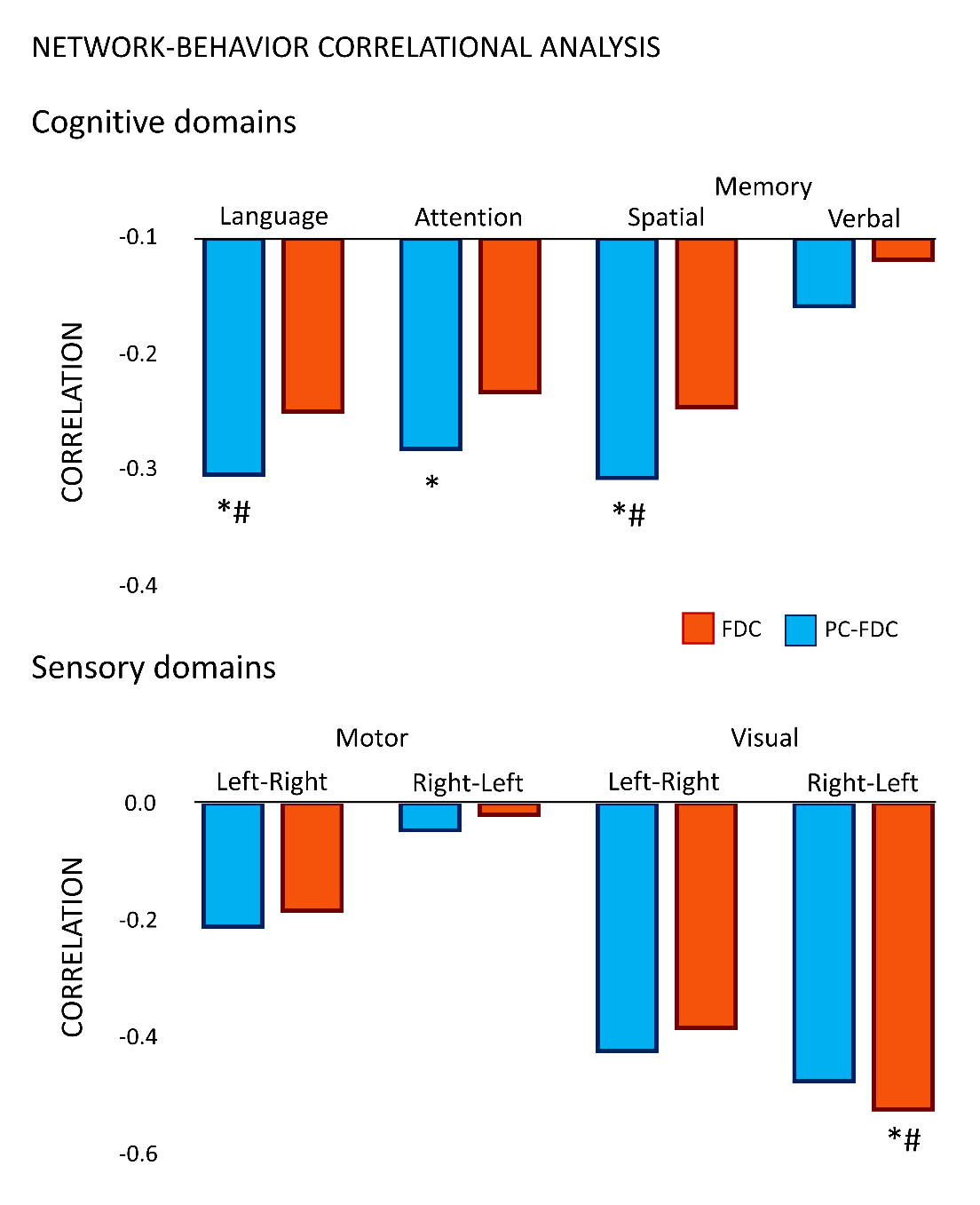
**

**Supplementary Figure S5.** **Connectivity and sensory domains.**

Univariate maps (threshold-free cluster enhancement approach with n=5000 permutation) for the visual and motor (left-right) domain are shown at a pFWE corrected threshold < 0.05. The corresponding dice coefficient with canonical template is shown for both approaches and domains (inset bar plots). Abbreviations: SMN: sensorimotor network; VIS: visual. L: left; R: right.

**
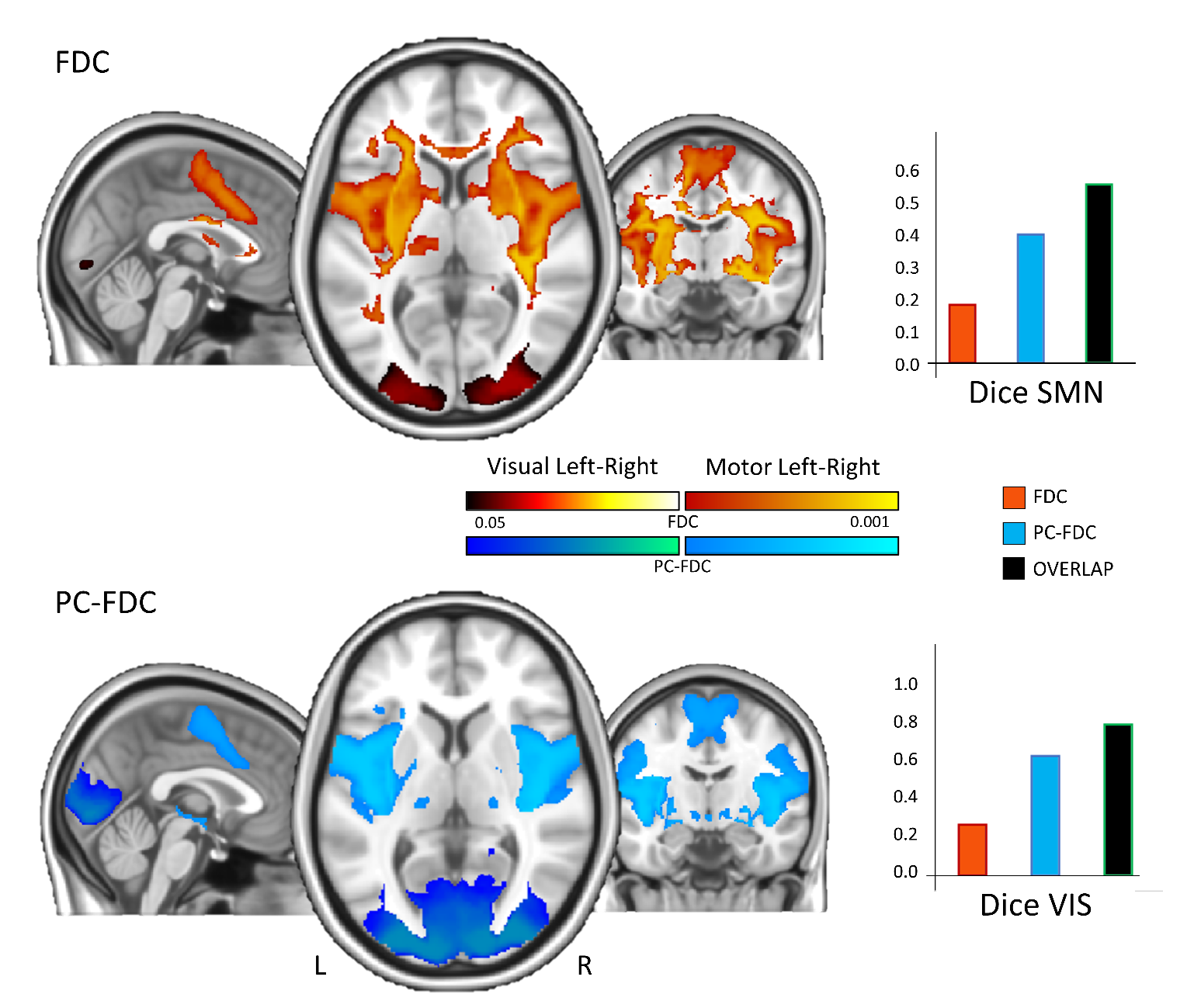
**

**Supplementary Figure S6.** **Overlap between univariate and ridge regression maps.**

Only maps showing significant clusters in the ride regression and the univariate approaches are reported. Univariate maps were threshold at p<0.01 FWE-corrected, except for the motor right-left and visual left-right, reported for comparison purpose at a less stringent threshold of p<0.05 FWE; ridge regression maps were gaussian smoothed (sigma=1) and linear normalized to improve visualization, and threshold at an arbitrary value of -0.2. Maps were overlaid to brain surface in MNI space.

**
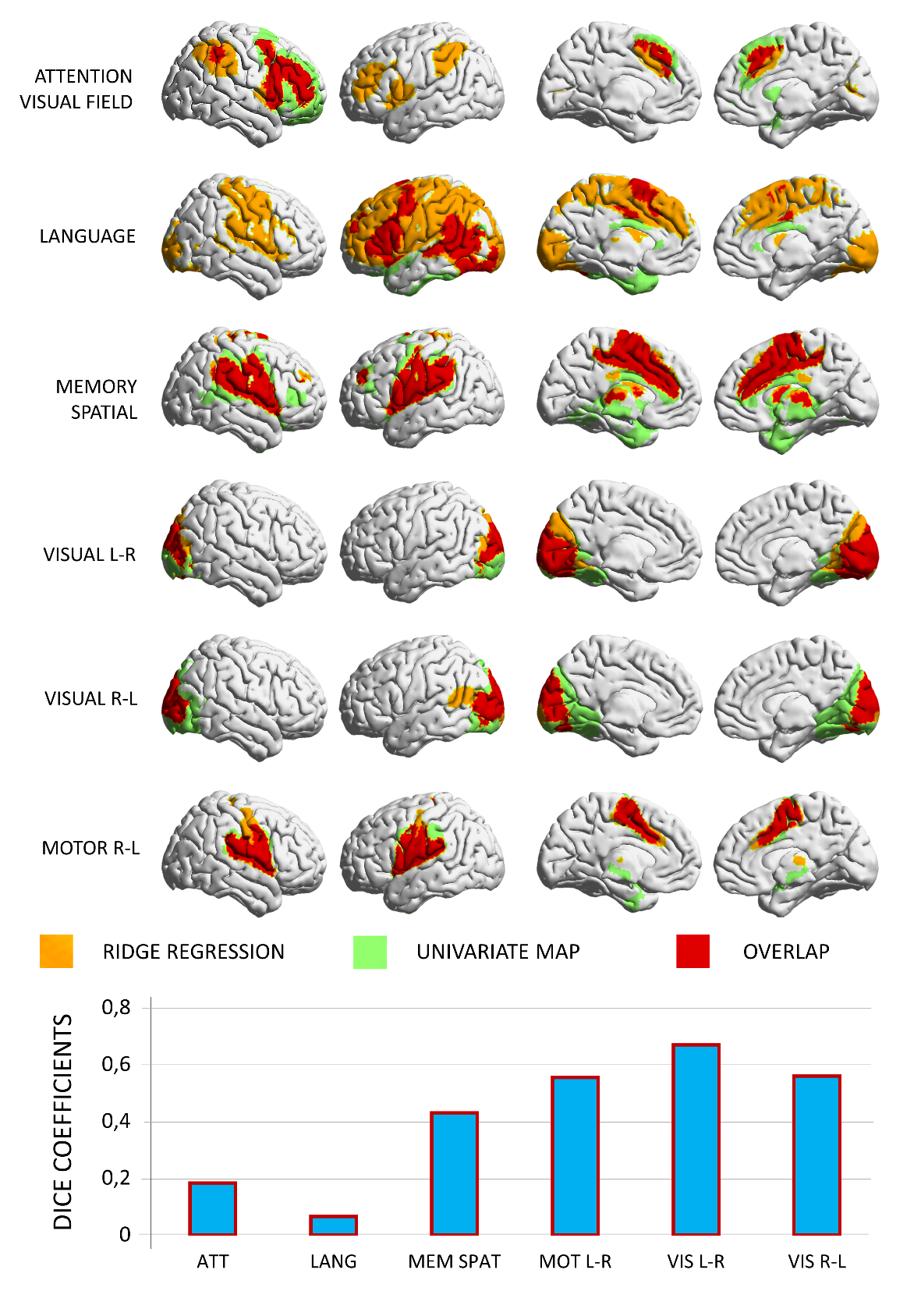
**

**Supplementary Figure S7. Comparison between PC-FDC maps at different thresholds**.

Network strength maps computed at different coefficients threshold. Different PCA coefficients showed significant differences in network strength (Friedman test: 90^th-CDF^ p < 0.001; 95^th-CDF^ p < 0.001; 99^th-CDF^ p < 0.001). Post-hoc analysis revealed that the 20^th^ PCA-percentile maps showed the highest connectivity values. CDF: cumulative function distribution.

**
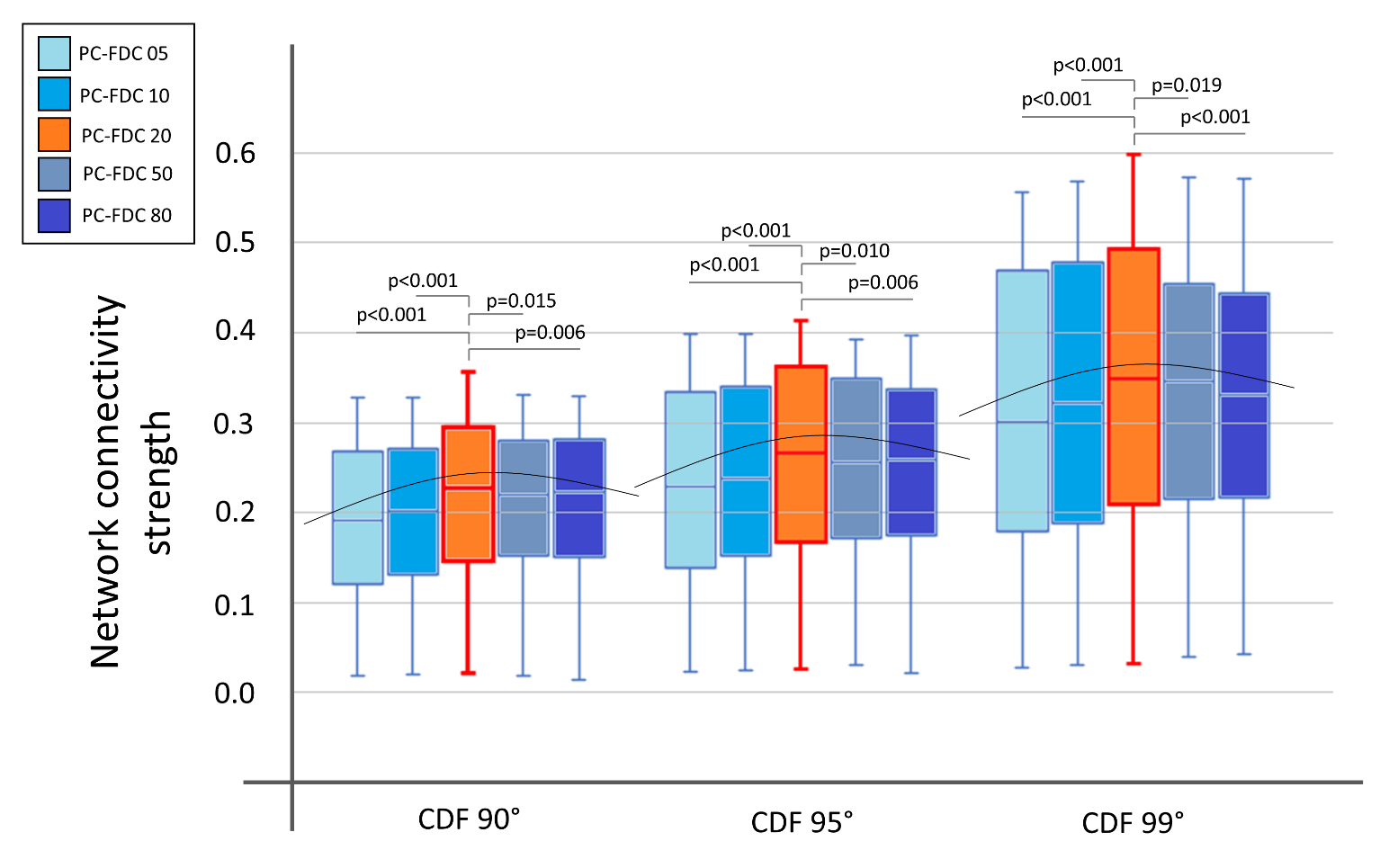
**

**Supplementary Figure S8. Comparison between PC1 and PC2.**

Panel A: amount of variance explained by the first and the second within-lesion PCs; Panel B: comparison between networks computed with the first and the second PCs of a representative lesion from the stroke sample.

**
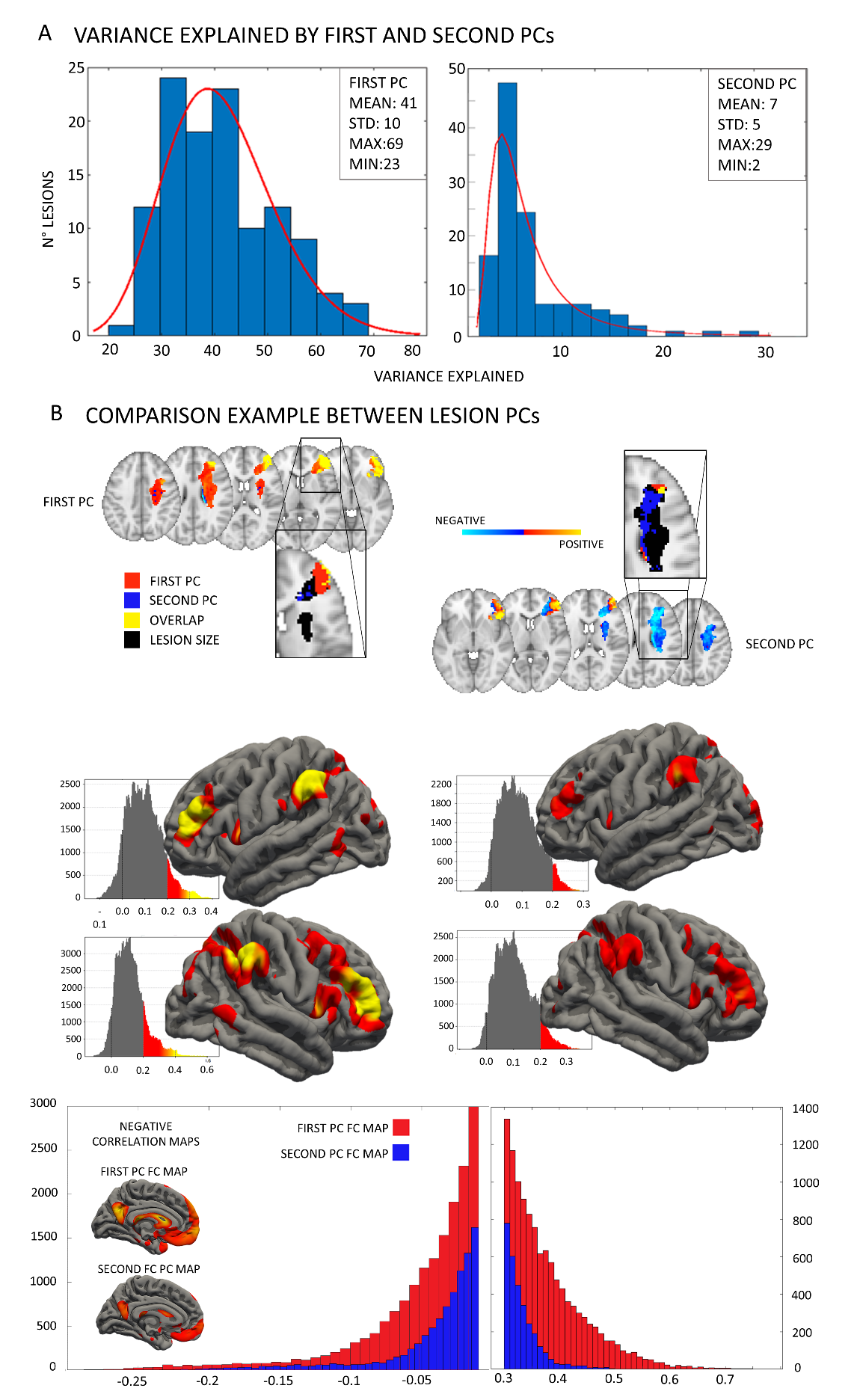
**
